# Feasibility of an unsupervised aerobic exercise training program for participants with persistent symptoms after SARS-CoV-2 infection

**DOI:** 10.1038/s41598-025-13905-4

**Published:** 2025-08-06

**Authors:** Carina Emmel, Oliver Bruder, Niklas Keller, Lukas Schipper, Christoph Schneider, Alexander Ferrauti, Mirjam Frank, Marcus Brinkmann, Mareike Eißmann, Thimo Wiewelhove, Börge Schmidt

**Affiliations:** 1 Institute for Medical Informatics, Biometry and Epidemiology, University Hospital of Essen, University of Duisburg-Essen, Hufelandstraße 55, 45122 Essen, Germany; 2 Department of Cardiology and Angiology, Elisabeth-Krankenhaus, Essen, Germany; 3 Ruhr University Bochum, Bochum, Germany; 4 Department of Fitness and Health, IST University of Applied Sciences, Düsseldorf, Germany; 5 Department of Training & Exercise Science, Ruhr University Bochum, Bochum, Germany

**Keywords:** Epidemiology, Infectious diseases, Quality of life, Rehabilitation

## Abstract

Aim of the randomized controlled pilot study was to investigate general feasibility in terms of adherence to a 12-week unsupervised aerobic exercise training program in participants with persistent symptoms (fatigue, concentration problems, breathing problems or headache) > 12 months after SARS-CoV-2 infection. Multiple symptom-related, psychosocial, spiroergometric and body composition parameters were collected at baseline and follow-up examination. The intervention group underwent a 12-week unsupervised aerobic exercise training program. The control group kept their regular physical activity. Both groups maintained an online training diary. Data was evaluated descriptively using intention-to-treat, per protocol and as-treated analysis. Feasibility of an unsupervised aerobic exercise training intervention was limited, as a large number of participants in the intervention group did not strictly adhere to the training plan. Therefore, statements about the efficacy were restricted. However, there was some weak indication for efficacy of the intervention to reduce self-rated severity of symptoms and to improve overall wellbeing. In the present study, an unsupervised aerobic exercise training intervention can be recommended only to a limited extend. The study results can be used to inform future studies designed to evaluate the efficacy of unsupervised aerobic exercise training programs in participants with persistent symptoms after SARS-CoV-2 infection.

**Supplementary Information:**

The online version contains supplementary material available at 10.1038/s41598-025-13905-4.

## Introduction

Globally, millions of people were infected with the severe acute respiratory syndrome coronavirus 2 (SARS-CoV-2), causing coronavirus disease 2019 (COVID-19)^1,2^. COVID-19 is now recognized as a multi-organ disease with a broad spectrum of manifestations^3^and is associated with substantial morbidity and mortality^1^.

Post COVID-19 condition, an important sequela of SARS-CoV-2 infection, is defined as symptoms that appear three months after the onset of the infection, either as new symptoms following initial recovery from an acute COVID-19 episode or as persisting symptoms from the initial illness, lasting for at least two months. Symptoms cannot be explained by an alternative diagnosis, may fluctuate or relapse and generally have an impact on everyday functioning^4^. A prevalence of 32–46% of COVID-19-related symptoms at three months^5–7^ and a prevalence of 41% at > 12 months post SARS-CoV-2 infection have been reported^6^. In a population-based study, 50.7% of study participants reported at least one COVID-19-related symptom at nine months post infection^8^. A variety of persistent symptoms in post COVID-19 condition were reported including fatigue, headache, attention disorder, hair loss, dyspnoea, cough, chest pain, myalgia, joint pain, impaired mobility, cognitive impairment, olfactory and gustatory dysfunction, sleep disorder, depression, anxiety^9^. The most commonly reported symptoms one year after the infection were fatigue, dyspnoea, sleep disorders and myalgia^5^. These symptoms decreased physical and mental health-related quality of life and could reduce independence in activities of daily living^7,9^.

Exercise plays an important role in the prevention, but also in the therapy of numerous diseases^10–12^. A protective effect of regular physical activity against a severe course of the disease in the case of an infection with SARS-CoV-2 has been suggested in retrospective observational studies. Persistent inactivity, on the other hand, may intensify COVID-19 related symptoms^13,14^. The guideline on Long-/Post-COVID of the German Society of Pneumology and Respiratory Medicine and other German societies recommended a controlled instruction in physical activity or a dosed physical training for patients with post COVID-19 symptoms such as fatigue, cough and concentration problems^15^. A recently published report and meta-analysis by Torres and Gradidge^16^ suggested that exercise rehabilitation interventions improved cardiorespiratory fitness and pulmonary function, functional and physical capacity and health related quality of life in patients with post COVID-19 condition. Various types of rehabilitation interventions were implemented in the studies reviewed, including aerobic exercise, flexibility, proprioception, breathing, respiratory exercise, muscular endurance exercise, gymnastic and balance. Telehealth and home-based exercise programs likewise demonstrated beneficial effects. The authors emphasized the need for future studies with robust randomised controlled trials, as most of the included studies in the report had no control group. Furthermore, as most of the studies investigated a multi-disciplinary approach to rehabilitation, more research on the effectiveness of specific, independent interventions with a detailed description on volume and pattern of progression was encouraged^16,17^. The need for quality reporting in exercise interventions in health and disease to replicate the exercise program in different clinical settings was highlighted in another recent systematic review (‘If exercise is medicine, why don’t we know the dose?’)^18^. Evidence on post COVID-19 condition > 12 months after infection remains limited, as most studies have investigated the condition 3 to 12 months after infection. Even though some studies investigated telehealth and home-based exercise programs, there is still limited knowledge on the effectiveness of unsupervised interventions, especially in specific, independent exercise interventions.

Objective of the Physical activity & post COVID-19 condition (SPOVID) randomized controlled pilot study was to investigate general feasibility of a 12-week unsupervised aerobic exercise training program in participants with persistent symptoms (fatigue, concentration problems, breathing problems or headache) of moderate severity > 12 months after infection with SARS-CoV-2. The intervention program aimed at meeting the World Health Organization physical activity guidelines per week^19^. Adherence to the intervention procedure was the primary feasibility outcome. Multiple symptom-related, psychosocial, spiroergometric and body composition parameters were collected at study baseline and at follow-up examination in order to quantify potential effects of the aerobic exercise training program. This was a strictly explorative pilot study using descriptive analyses to inform future research testing the efficacy of an unsupervised aerobic exercise training program (i.e., no hypotheses were stated, no group differences were tested, no outcome efficacy cut-offs were provided, no formal a priori feasibility thresholds, e.g., for recruitment, retention, adherence were defined).

## Methods

### Study design and study population

The SPOVID pilot study was a randomized controlled intervention study (parallel group design) embedded in the POSTCOVE study, a prospective cohort study of 800 participants. The POSTCOVE study aimed to assess persistent symptoms, overall health status and (sub-)clinical markers for cardiovascular, metabolic and respiratory conditions in participants > 12 months after SARS-CoV-2 infection. Recruitment for the POSTCOVE cohort took place between September 2021 and May 2023). Eligible participants for POSTCOVE were residents of the City of Essen, Germany, aged 18–75 years, who had been registered by local health authorities as having tested positive for SARS-CoV-2 infection, with a date of first infection between February 2020 (the beginning of the first pandemic wave in Essen) and November 2020.

The SPOVID pilot study was explorative in nature; therefore, no formal sample size calculation was performed. Based on the assumption that approximately 10% of the POSTCOVE cohort would meet the specific inclusion criteria for the SPOVID pilot study (see below), it was assumed feasible to recruit 60 participants during the first ~ 9 month of the POSTCOVE recruitment period. This number of participants has been described as being adequate for pilot studies with the aim of assessing potential feasibility problems with a moderate to high prevalence^20^.

**Fig. 1:**
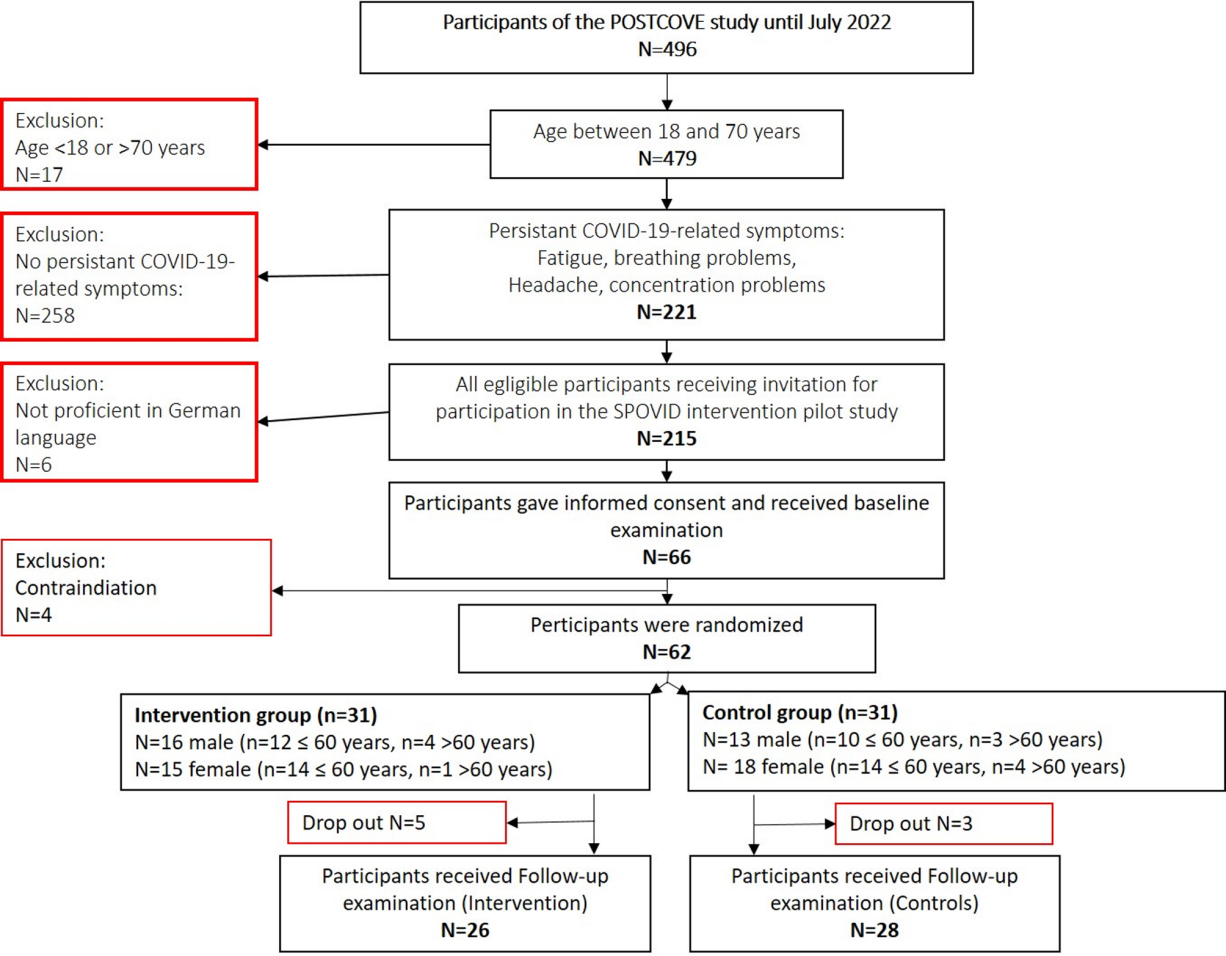
Flow chart of study population.

From the POSTCOVE cohort, all participants aged between 18 and 70 years (*n* = 479) who had completed study examination by July 22, 2022, were then screened for at least one of the self-reported, on-going COVID-19-related symptoms: fatigue, concentration difficulties, breathing problems or headache (see Fig. 1). Of these, participants with sufficient German language proficiency (*n* = 215) were invited to participate in the SPOVID pilot intervention study. 66 (30.7%) individuals were willing to participate, gave informed consent and underwent their baseline examination (T0) at the Department of Cardiology and Angiology, Elisabeth Hospital in Essen, between May and August 2022. This time frame was chosen to enable all participants to begin the intervention during late spring or summer.

At T0, participants received a comprehensive internistic-cardiological health assessment to evaluate general physical resilience and identify potential clinical contraindications for participation in a structured physical intervention program. Further exclusion criteria included a current SARS-CoV-2 infection; being classified as a trained or higher-level athlete according to the participant classification framework proposed by McKay et al. (2022)^21^; chronic fatigue syndrome; pregnancy or breastfeeding; current in-patient treatment; or unwillingness to begin and consistently adhere to an exercise training program during the study period. If any contraindications were identified, participants were not randomized and were referred for further medical evaluation. Four participants were excluded due to cardiovascular problems. None of the included participants met diagnostic criteria for chronic fatigue syndrome, as none reported post-exertional malaise in particular and all expressed confidence in their ability to follow a regular training program.

Of the 66 participants who gave informed consented to participate in the SPOVID pilot study, 62 met al.l inclusion criteria and had no medical contradictions (Fig. 1). These participants were individually randomized in a 1:1 ratio to either the intervention or control group, stratified by sex and age group (≤ 60 years/>60 years). Randomization was performed using the online service ALEA^22^which employs the minimisation technique described by Pocock and Simon^23^ that minimizes imbalance in the distributions of treatment numbers within the levels of each individual stratification factor.

A sport scientist blinded to the results of the medical examination, entered the relevant participant information (sex and age) into the ALEA software tool to determine the random group allocation to then generate an enrolment form containing the participant’s data and allocation results. A total of 31 participants were allocated to the intervention group and 31 to the control group (Fig. 1).

All enrolment forms were securely stored in a folder accessible only to the sport scientist responsible for randomisation. While the sport scientist guiding the intervention and supervising participants, as well as the participants themselves and the data analyst, were aware of the group allocation results, the medical staff conducting the physical examination- including spiroergometry, body composition examination and face-to-face interviews, were blinded to group allocation. Participants were instructed not to reveal their group allocation during the follow-up examination (T1).

T1 examination took place 12 weeks after baseline to assess the effects of the exercise intervention.

The intervention phase and all follow-up examinations were completed by December 2022.

A total of eight participants (*n* = 5 from the intervention group, *n* = 3 from the control group) dropped out due to the following reasons: time constraints related to training adherence or diary documentation (*n* = 2), personal reasons (*n* = 1), relocation (*n* = 1), inability to participate in the T1 examination (*n* = 2), injury unrelated to the intervention program (*n* = 1), and acute illness unrelated to the intervention program (*n* = 1). Consequently, 54 participants completed the T1 examination (Fig. 1).

The study was conducted in accordance with the guidelines and recommendations for ensuring Good Epidemiological Practice^24^approved by the ethics committee of the University Duisburg-Essen (approval number: 22-10565-BO), and monitored throughout its course. The study was performed in accordance with the ethical principles of the Declaration of Helsinki. The study was registered with the German Ministry of Education and Science prior to its start (#FKZ 01EP2104A/B; https://www.gesundheitsforschung-bmbf.de/de/spovid-sport-long-covid-syndrom-14348.php; registration date: January 12, 2021).

### Aerobic exercise training program

The intervention group underwent a 12-week, unsupervised aerobic exercise training program focused on low intensities aligning with zone two and three of a five-zone intensity scale^25^. Individual training zones, along with the corresponding heart rates, were determined based on ventilatory thresholds derived from an incremental exercise test. Training zones two and three and their corresponding exercise doses were positioned below the first ventilatory threshold and between the first and second ventilatory thresholds, respectively. Prior to training initiation, the exercise group received guidance on setting aerobic exercise intensity by the same specially trained sport scientist in a single two-hour session. Subsequently, participants were instructed to independently engage in running or walking-based aerobic exercise sessions three times a week, in accordance with their individual fitness levels and corresponding training zones. Although some participants reported engaging in regular physical activity prior to enrolling in the study (Table 1), the training program still represented an increase in systematically planned, training-related physical activity for all participants. To enhance engagement and training effectiveness, the program included a blend of steady-state and interval training, along with progressive weekly increases in both training volume and intensity. Every fourth week was designed a recovery week with reduced training volume, establishing an undulatory loading scheme (Supplementary Table S1). Bi-weekly telephone check-ins were utilized for tracking progress throughout the training period.

**Table 1:** Characteristics of intention-to-treat analysis population at baseline examination (T0).

Parameter name (unit)	Intervention group	Control group
N	*n* = 26	*n* = 28
Female Sex^#^	11 (42.3%)	16 (57.1%)
Age (years)* [n_miss_ = 0]	51.6 (11.9 ± SD)	52.4 (9.8 ± SD)
Weeks between SARS-CoV-2 infection and baseline examination ^†^ [n_miss_ = 0]	101.3 (91.6–114.6)	99.1 (95.1–112.6)
Weeks between baseline and follow-up examination ^†^ [n_miss_ = 0]	13.4 (13.1–14.3)	12.9 (12.1–14.9)
SARS-CoV-2 re-infection before baseline examination^#^ [n_miss_ = 2]	5 (20.8%)	10 (35.7%)
Sport activity before SARS-CoV-2 infection^#^ [n_miss_ = 2]	21 (87.5%)	21 (75.0%)
Performance of training after SARS-CoV-2 infection ^#^ [n_miss_ =3 ]	19 (79.2%)	19 (70.4%)
Former sport activities^#^ [n_miss_ = 6]		
Regular	17 (68.0%)	11 (47.8%)
Occasional	5 (20.0%)	9 (39.1%)
Rare	3 (12.0%)	1 (4.4%)
Never	0 (0.0%)	2 (8.7%)
Former competitive athletes ^#^ [n_miss_ = 2]	7 (29.2%)	6 (21.4%)
Smoking status^#^ [n_miss_ = 0]		
Current	2 (7.7%)	4 (14.3%)
Former	9 (34.6%)	10 (35.7%)
Never	15 (57.7%)	14 (50.0%)
Previous or current comorbidities		
Arterial hypertension ^#^ [n_miss_ = 0]	4 (15.4%)	6 (21.4%)
Diabetes mellitus Type II^#^ [n_miss_ = 0]	1 (3.9%)	0 (0.0%)
Dyslipoproteinemia^#^ [n_miss_ = 0]	1 (3.9%)	2 (7.1%)
Asthma and/or allergy^#^ [n_miss_ = 0]	11 (42.3%)	17 (60.7%)
Cardiovascular disease^#^ [n_miss_ = 0]	4 (15.4%)	8 (28.6%)
Metabolic disease^#^ [n_miss_ = 0]	3 (11.5%)	3 (10.7%)
Other lung disease^#^ [n_miss_ = 0]	2 (7.7%)	6 (21.4%)
Neurological disease^#^ [n_miss_ = 0]	1 (3.9%)	2 (7.1%)
Mental disease^#^ [n_miss_ = 0]	6 (23.1%)	2 (7.1%)
Gastrointestinal disease^#^ [n_miss_ = 0]	2 (7.7%)	4 (14.3%)
Orthopaedic disease^#^ [n_miss_ = 0]	14 (53.9%)	13 (46.4%)
Cancer	2 (7.7%)	0 (0.0%)
Other disease^#^ [n_miss_ = 0]	7 (26.9%)	4 (14.3%)
Surgery^#^ [n_miss_ = 0]	14 (53.9%)	20 (71.4%)
Accident^#^ [n_miss_ = 0]	4 (15.4%)	8 (28.6%)
Hospitalization^#^ [n_miss_ = 0]	14 (53.9%)	12 (42.9%)

*Mean ± standard deviation (SD), ^#^ proportion (%), ^†^ median (interquartile range), n_miss_ number of participants with missing values.

Participants in the control group were asked to maintain their habitual physical activity patterns between baseline (T0) and follow-up (T1) and were offered the training intervention program after the T1 examination. All participants (intervention and control) documented their training adherence and additional physical activity using an online training diary (REGmon^26^.

### Parameters

At T0 and T1, standardised face-to-face interviews with a cardiologist were conducted to assess the following clinical parameters: persistent self-perceived symptoms > 12 months after SARS-CoV-2 infection (fatigue, concentration difficulties, headache, dyspnea) using a 0–10 numeric scale (10 = highest intensity) the intensity related to their SARS-CoV-2 infection was measured, SARS-CoV-2 re-infection prior to T0 examination, current physical performance rated on a 0–10 scale (0 representing “very restricted” and 10 representing “very powerful”). Following previous or current comorbidities and risk factors such as past surgeries, accidents and hospitalizations, present disability were collected: cardiovascular, metabolic, neurological, mental, gastrointestinal, orthopaedic and other lung disease, asthma and/or allergies, arterial hypertension, diabetes mellitus, dyslipoproteinemia and smoking status (categorized as current, former or never).

At T1, prevalent diseases, accidents, surgeries and disabilities occurring between T0 and T1 were assessed.

At T0, a separate standardized face-to-face interview with a sport scientist collected data on prior sporting activities and post-infection exercise behaviour. Information on current health status (assessed on a 1–5 scale, 1= “very good” to 5= “poor”), quality of life (assessed on a 1–4 scale with 1= “very bad” to 4= “good”), satisfaction with life in general and with health (assessed on a 1–4 scale with 1= “very dissatisfied” to 4=“very satisfied”), and sleep quality in the past month (assessed on a 1–3 scale with 1=“very good” to 3=“very bad”). Further, depressive symptoms were assessed using the 15-item Centre for Epidemiologic Studies Depression Scale (CES-D) with a range of 0 to 45 points, higher scores indicating greater symptom burden^27^. Also, physical activity-related health competence (PAHCO) was rated using a the PAHCO- questionnaire consisting of 42-items (condensed into 10 first-order scales and additionally pooled into three second-order scores representing movement competence, control competence and self-regulation competence)^28^. Symptoms of depression and physical activity-related health competence were collected at T0 and T1 in standardized and validated self-administered questionnaires. Mean scores of the three second-order scores of the PAHCO-questionnaire with the range of 1 to 4 were used in the analysis, with higher values indicating higher competence.

The internistic-cardiological health check to determine general physical resilience and potential clinical contraindications included the following examinations: vital parameter status (heart rate, blood pressure, respiratory rate), auscultation of the heart, abdominal palpation, presence of edema or bowel sounds, NYHA-classification to categorize heart failure, transthoracic echocardiography, 12-channel ECG and bodypletysmography to assess pulmonary function. Laboratory analyses were performed to determine a complete blood count and to collect several additional biomarkers such as Troponin T, pro-BNP, e-CRP and D-Dimere and 25-OH-Vit.-D status. Parameters of the bioelectrical impedance analysis and spiroergometry on the treadmill ergometer including exercise ECG were analysed to assess the influence of the exercise training program. In the bioelectrical impedance analysis height, weight, body mass index, skeletal muscle mass and body fat were determined with a standard stadiometer and a biometrical impedance analysis system (InBody Deutschland, Eschborn, Germany). To determine peak oxygen consumption ($\dot {V}$O2peak), peak power output (Wpeak), peak heart rate (HRpeak), peak respiratory exchange ratio (RERpeak), as well as W and HR at ventilatory thresholds 1 (VT1) and 2 (VT2), W and HR at lactate thresholds, and heart rate recovery during the first 3 min after test cessation (HRR2), a stepwise incremental cycle ergometer test was conducted using a Cyclus 2 ergometer (RBM elektronik-automation GmbH, Leipzig, Germany). The test involved spiroergometry, exercise electrocardiography (ECG), and blood lactate diagnostics. Commencing with an initial resistance of 50 W, resistance increased by 25 W every 3 min until subjective exhaustion. Participants were free to cycle at their preferred pedal rate, as the Cyclus 2 ergometer maintains a constant power condition independent of pedal cadence.

Gas exchange data were continuously collected using a breath-by-breath gas collection system (Metalyzer 3B, Cortex Biophysik GmbH, Leipzig, Germany). Gas calibrations were performed before each test in accordance with the manufacturer’s instructions. A rolling average over 30 s was applied for respiratory data smoothing, and the highest 30-second rolling averages during the test were defined as $\dot {V}$O2peak and RERpeak. Wpeak was calculated as follows: Wpeak = Wf + [(t/D x P)], where Wf represents the value of the last completed workload (W), t is the time (s) the last uncompleted workload was sustained, D is the duration (s) of each stage, and P is the power output difference between workloads. W and HR at VT1 and VT2 were determined visually by combining four methods: the ventilatory equivalent method, the excess carbon dioxide method, the V-slope method, and the end-tidal method. Two trained sports scientists independently and blindly assessed each participant’s graphic data, followed by a conference to reconcile any differences and arrive at a consensus for each threshold.

HR was monitored and recorded during the test via a 12-lead ECG (custo cardio 100/ERG BT, custo med GmbH, Ottobrunn, Germany), and HRpeak as well as HRR after the test were determined from the data. Capillary whole-blood samples were obtained from the earlobe before the test, during the last 15 s of each stage, and at the point of exhaustion. These samples were analyzed for lactate (La) using 20-µL capillaries, hemolyzed in 1-mL microtest tubes, and subjected to amperometric-enzymatic analysis using the Biosen C-Line Sport (EKF-diagnostic GmbH, Barleben, Germany). From the resulting lactate values, W and HR at aerobic (2 mmol/l) and anaerobic lactate thresholds (4 mmol/l) were determined, following the methodologies outlined by Mader et al.^29^ and Heck et al.^30^.

### Training diary

Participants from both the intervention and the control group maintained an online training diary (REGmon)^26^which served as a tool for tracking their adherence to the training plan and to document any training that was completed outside the study conditions. The digital platform enabled participants to log both external and internal training loads, including the date of the training session, the type of activity (such as jogging, walking, cycling, swimming, or rowing), training volume, and training intensity. Training duration was documented in terms of time spent on each exercise session, while mean training intensity was rated by the subjects following each training session using a 10-point category-ratio (CR-10) rating of perceived exertion (RPE) scale^31–33^. The internal training load for each training session was calculated using the session rating of perceived exertion (session-RPE) method^32,34^. This method involved multiplying the absolute training duration in minutes by the training intensity. The online training diary also served as a repository for recording significant events that could affect training progress. Participants documented occurrences such as injuries, illness, or other relevant circumstances that might influence their training progression.

### Description of the Intention-to-treat, per-protocol and as-treated analysis population

In the ITT analysis, all participants with non-missing information (*n* = 54) were included according to their randomization (*n* = 26 in intervention group, *n* = 28 in control group) (Table 2). The PP population consisted exclusively of participants without major protocol deviation within their assigned group defined as at least 27 documented training sessions within training zone two and three of the five-zone intensity scale^25^ for intervention group participants and less than 18 documented training sessions for control group participants. 3 participants from the intervention group with 18–26 exercise sessions at training zone two and three had to be excluded, because they were not concordant with either of the two groups. Likewise, 2 participants from the intervention group with a stop of training due to documented infection or injury for more than 6 weeks (out of the 12 weeks training program) as well as 6 participants (*n* = 4 of the intervention group, *n* = 2 of the control group) with SARS-CoV-2 re-infection during T0 and T1 were excluded from PP analysis. This resulted in a per-protocol population of 37 participants (*n* = 14 in intervention group, *n* = 23 in control group) (Table 2). The AT analysis classified participants according to the actual training they reported rather than the study group they were assigned to applying the same definition of training volume and intensity for the intervention and control group as in PP population. Accordingly, 3 participants randomized to the intervention group reporting less than 18 exercise sessions were assigned to the control group. Conversely, 3 participants randomized to the control group reporting more than 27 exercise sessions within training zone two or three were assigned to the intervention group. This resulted in an AT population of 43 participants (*n* = 17 in intervention group, *n* = 26 in control group) (Table 2).

**Table 2:** Description of the population size of the intention-to-treat, per-protocol and as-treated analysis population.

	Intention-to-treat	Per-protocol	As-treated
Intervention group	26	14	17
Control group	28	23	26
Sum	54	37	43

### Statistical analyses

Adherence to the intervention procedure was the primary feasibility outcome. This was a strictly explorative pilot study using descriptive analyses. No hypotheses were stated, no group differences were tested, no outcome efficacy cut-offs were provided, no formal a priori feasibility thresholds were defined. Data was evaluated using intention-to-treat (ITT, *n* = 26 intervention and *n* = 28 control group), per protocol (PP, *n* = 14 intervention and *n* = 23 control group) and as-treated (AT, *n* = 17 intervention and *n* = 26 control group) analysis (Table 2). As this study was designed as a feasibility study and there was no primary endpoint, all parameters that could be potentially affected by the training intervention were explored in separate statistical models to assess direction and strength of group differences in parameter change between T0 and T1. In ITT, PP and AT analysis mean values and standard deviations (SD) were generated for the intervention and control group at T0 and T1. Scores and Likert-scale categorical parameters were treated as continuous parameters in the analyses. Furthermore, the difference of mean values at the time of T1 subtracted by T0 was calculated separately for the intervention and control group, to assess the change in the respective parameter over time in each group. Additionally linear regression models for each parameter were fitted including a time-dependent dummy variable (1 = T1, 0 = T0), a treatment group dummy variable (1 = intervention group, 0 = control group) and an interaction term between time-dependent and treatment group dummy variables to calculate the difference in differences (DID) with corresponding 95% confidence interval (95%-CI). The DID indicates the difference in change of the intervention group compared to the control group over time. Taking the different response scale widths (number of scale points ranged from 3 to 46 points) of the analysed parameters into account and for better comparability of the magnitude of effect sizes across parameters, effect parameters and confidence intervals were described per standard deviation (SD) by dividing the DID by the SD of the respective parameter at T0 in the ITT analysis population. Due to the different response scale directions (some descending from “very good” to “bad” and others ascending) positive DID did not automatically represent indication of a stronger improvement in the intervention group. Therefore, the direction of the treatment effect was reported with a “+” in the last column of each table to intuitively spot when the direction of DID went in the direction of the hypothesis (i.e., stronger improvement in the intervention group). Clopper-Pearson 95% confidence intervals were calculated in order to assess whether the proportion of DID consistent with the hypothesis could be expected by chance^35^. Mean values and difference of mean values were calculated using SAS^®^ software v9.04^36^ and (SD-scaled) difference in difference with 95%-CI were calculated in R v4.1.2^37^.

### Direction of difference in differences (DID)

Direction of difference in differences (DID) was indicated with a “+” when direction of effect consistent to a positive DID ((T1-T0 intervention group) - (T1-T0 control group)) with a stronger positive difference (T1-T0) in the intervention group for parameters where higher values represent better health/wellbeing/fitness or consistent to a negative DID with a stronger negative difference in the intervention group for parameters where lower values represent better health/wellbeing/fitness and “-“ when direction of effect neither consistent to a positive DID ((T1-T0 intervention group) - (T1-T0 control group)) with a stronger positive difference (T1-T0) in the intervention group for parameters where higher values represent better health/wellbeing/fitness nor consistent to a negative DID with a stronger negative difference in the intervention group for parameters where lower values represent better health/wellbeing/fitness).

## Results

### Description of study population

Table 1 describes the characteristics of the ITT analysis population at T0 stratified by study group (Table 1). Intervention and control group consisted of 42% and 57% female participants, respectively. Mean age was 52 years in both groups. A median of 101.3 and 99.1 weeks between SARS-CoV-2 infection and T0 examination was observed for the intervention and control group, respectively. The average time between T0 and T1 examination was slightly higher in the intervention group. Supplementary Figure S1 displays the time between T0 and T1 for each participant stratified by group. Overall, few participants showed strong deviation from the target time of 12 weeks between examinations with more extreme values in the control group. The strongest deviations (16.5, 17.0, 20.9 and 21.1 weeks) in the intervention group were the consequence of documented acute illness (SARS-CoV-2 re-infection and influenza) and injuries for the duration of 2 to 4 weeks during or at the end of the exercise training period. In one case, no training was recorded because of an injury.

A number of 5 participants (21%) in the intervention group and 10 participants (36%) in the control group documented a SARS-CoV-2 re-infection before T0 examination (Table 1). Most participants performed regular (68% intervention group, 48% control group) or occasional (20% intervention, 39% control group) former sport activities and 79% in the intervention group and 70% in the control group reported sport activities after their first SARS-CoV-2 infection. There were 7 former competitive athletes in the intervention and 6 in the control group. Mean baseline self-rated quality of life and satisfaction with health were between fair to good, physical activity-related health competence (movement, control and self-regulation competence), SARS-CoV-2-related symptoms, current performance and sleep quality were self-rated as intermediate (Table 3).

**Table 3:** Baseline (T0) and follow-up (T1) means and standard deviation (SD) in intervention and control group, difference between T1 and T0 within groups, difference in differences of intervention and control group with corresponding 95% confidence intervals (95%-CI) of self-rated symptom-related and psychosocial parameters in intention-to-treat analysis.

Parameter name (range)	Intervention baseline (T0) Mean ± SD	Control baseline (T0) Mean ± SD	Intervention follow-up (T1) Mean ± SD	Control follow-up (T1) Mean ± SD	Difference intervention T1-T0	Difference control T1-T0	Difference in differences (DID) T1-T0 [95%-CI]	Difference in differences (DID) T1-T0 [95%-CI} standardized^§^	Direction of DID^$^
N	26	28	26	28					
Current health status (1–5)	2.88 ± 0.77	2.86 ± 0.76	2.54 ± 0.81	2.78 ± 0.89	− 0.35	− 0.08	− 0.27 [− 0.89,0.35]	− 0.35 [− 1.18,0.47]	+
Quality of life (1–4)	3.50 ± 0.58	3.50 ± 0.51	3.62 ± 0.57	3.59 ± 0.57	0.12	0.09	0.02 [− 0.41,0.45]	0.03 [− 1.18,0.47]	+
Satisfaction in general (1–4)	3.23 ± 0.59	3.21 ± 0.57	3.04 ± 0.72	3.00 ± 0.68	− 0.19	− 0.21	0.02 [− 0.47,0.51]	0.04 [− 0.82,0.90]	−
Satisfaction with health (1–4)	3.08 ± 0.63	3.04 ± 0.51	3.12 ± 0.59	3.23 ± 0.71	0.04	0.20	− 0.16 [− 0.63,0.31]	− 0.28 [− 1.11,0.56]	−
Movement competence* (1–4)	2.90 ± 0.67	2.62 ± 0.87	3.07 ± 0.62	2.68 ± 0.74	0.17	0.06	0.12 [− 0.45,0.69]	0.15 [− 0.57,0.87]	+
Control competence* (1–4)	2.62 ± 0.70	2.40 ± 0.62	2.87 ± 0.62	2.50 ± 0.60	0.25	0.10	0.15 [− 0.34,0.64]	0.23 [− 0.52,0.97]	+
Self-regulation competence* (1–4)	2.81 ± 0.61	2.80 ± 0.63	2.91 ± 0.60	2.65 ± 0.67	0.11	− 0.16	0.26 [− 0.23,0.75]	0.43 [− 0.37,1.22]	+
Symptoms of depression (0–45)	9.64 ± 6.39	10.14 ± 7.95	8.43 ± 7.32	10.89 ± 7.18	− 1.21	0.75	− 1.96 [− 7.55,3.63]	− 0.27 [− 1.05,0.51]	+
Fatigue^#^ (0–10)	4.81 ± 1.94	4.70 ± 2.05	3.76 ± 2.02	4.40 ± 2.16	− 1.04	− 0.30	− 0.75 [− 2.53,1.03]	− 0.38 [− 1.28,0.52]	+
Concentration disorders^#^ (0–10)	4.41 ± 2.12	4.04 ± 2.34	3.53 ± 1.92	3.86 ± 2.38	− 0.88	− 0.18	− 0.70 [− 2.76,1.36]	− 0.31 [− 1.24,0.61]	+
Difficulty breathing^#^ (0–10)	4.40 ± 1.96	4.41 ± 2.29	3.33 ± 1.87	3.83 ± 1.92	− 1.07	− 0.58	− 0.49 [− 2.58,1.60]	− 0.23 [− 1.22,0.76]	+
Headache^#^ (0–10)	4.57 ± 2.82	4.10 ± 2.51	2.00 ± 1.26	4.07 ± 2.69	− 2.57	− 0.03	− 2.54 [− 6.05,0.98]	− 0.99 [− 2.36,0.38]	+
Current physical performance (0–10)	5.46 ± 1.77	5.39 ± 1.89	6.77 ± 1.66	5.50 ± 1.82	1.31	0.11	1.20 [− 0.17,2.57]	0.66 [− 0.09,1.41]	+
Sleep quality (1–3)	2.12 ± 0.59	2.30 ± 0.54	2.04 ± 0.60	2.19 ± 0.63	− 0.08	− 0.10	0.03 [− 0.43,0.48]	0.05 [− 0.76, 0.86]	−

Current health status (1–5 scale with 1 representing “very good” and 5 “bad”), quality of life (1–4 scale with 1 meaning “very bad” and 4 “good”), satisfaction with life in general (1–4 scale with 1 representing “very dissatisfied” and 4 “very satisfied”), satisfaction with health (1–4 scale with 1 meaning “very bad” and 4 “good”), *Sub-competence of physical activity-related health competence (by Pfeifer et al. 2013) presented in mean scale (higher value indicating higher competence), symptoms of depression rated by 15-item Centre for Epidemiologic Studies Depression Scale (CES-D) with a cut-off value of > 17 representing depressive conditions, ^#^current symptoms related to SARS-CoV-2 infection (higher values indicating stronger impairment), current physical performance (0–10 scale with 0 representing “very restricted” and 10 representing “very powerful”), sleep quality ( 1–3 scale with 1 meaning “very good” and 3 “very bad”), ^§^ Difference in difference (DID) per standard deviation (SD) of the analysis population at study baseline (T0), ^$^ “+” means direction of effect consistent to a positive DID ((T1-T0 intervention group) - (T1-T0 control group)) with a stronger positive difference (T1-T0) in the intervention group for parameters where higher values represent better health/wellbeing/fitness or consistent to a negative DID with a stronger negative difference in the intervention group for parameters where lower values represent better health/wellbeing/fitness and “−” direction of effect consistent to a positive DID ((T1-T0 intervention group) - (T1-T0 control group)) with a stronger positive difference (T1-T0) in the intervention group for parameters where higher values represent better health/wellbeing/fitness or consistent to a negative DID with a stronger negative difference in the intervention group for parameters where lower values represent better health/wellbeing/fitness.

Orthopaedic diseases were the most reported comorbidities (previous or current) at T0 with 54% in the intervention and 46% in the control group. The largest difference between groups was noted for asthma and allergies (42% vs. 61%) (Table 1). Acute diseases reported to be most prevalent between T0 and T1 examination were cardiovascular and orthopaedic diseases (Table 4). 4 participants of the intervention group and 2 participants of the control group indicated a SARS-CoV-2 re-infection during the intervention period. More surgeries and hospitalizations not related to the exercise training program occurred in the intervention group between T0 and T1 compared to the control group (Table 4).

**Table 4:** Disease, surgery, accident and hospitalization of intention-to-treat analysis population reported to be prevalent between baseline (T0) and follow-up examination (T1).

Parameter name (unit)	Intervention	Control
N	26	28
SARS-CoV2 re-infection^#^ [n_miss_ = 1]	4 (15.4%)	2 (7.1%)
Cardiovascular disease^#^ [n_miss_ = 0]	5 (19.2%)	1 (3.6%)
Metabolic disease^#^ [n_miss_ = 0]	1 (3.9%)	2 (7.1%)
Other lung disease^#^ [n_miss_ = 0]	2 (7.7%)	1 (3.6%)
Neurological disease^#^ [n_miss_ = 0]	0 (0%)	3 (10.7%)
Mental disease^#^ [n_miss_ = 0]	1 (3.9%)	1 (3.6%)
Gastrointestinal disease^#^ [n_miss_ = 0]	1 (3.9%)	1 (3.6%)
Orthopaedic disease^#^ [n_miss_ = 0]	6 (23.1%)	5 (17.9%)
Other disease^#^ [n_miss_ = 0]	3 (11.5%)	0 (0%)
Surgery^#^ [n_miss_ = 0]	4 (15.4%)	2 (7.1%)
Accident^#^ [n_miss_ = 0]	2 (7.7%)	1 (3.6%)
Hospitalization^#^ [n_miss_ = 0]	6 (23.1%)	1 (3.6%)

^#^Proportion (%).

### Description of training diary data

Planned quantitative training load data (i.e., number of training weeks, total training sessions, weekly training sessions, total training duration, weekly training duration, average training intensity, total training load, and weekly training load) and actual mean (± SD) training load data of the intervention group and the control group are presented in Supplementary Table S2-S4. In the ITT analysis, both the total average training load (duration x intensity, determined by using the session rating of perceived exertion (session-RPE) method^32^ of the intervention group and the total average training load of the control group were slightly higher than the planned training load for the intervention group. In the PP analysis and, ultimately, in the AT analysis, larger differences in quantitative training load data were observed between the intervention group and the control group. The average quantitative training load data in the PP analysis and, especially, in the AT analysis was clearly higher in the intervention group than the planned training load. In contrast, the control group’s average quantitative training load was notably below the planned training load for the intervention group. However, even though the mean training load data corresponded to the planned training load data, there were substantial SDs in all training load data (i.e., sessions, duration, intensity and training load) in all analysis groups, indicating that a large number of participants did not strictly adhere to the training plan. For example, only 8 (out of 26) of the intervention group participants in the ITT analysis and 10 (out of 17) of the intervention group participants in the AT analysis documented 36 training sessions or more in total according to the training diary entries. Likewise, only 13 of the intervention group participants in the AT analysis reached the planned total training loads. On the other hand, there were few participants highly exceeding the planned training sessions and total training load, resulting in high mean values in the descriptive training load data.

### Compliance

Insufficient adherence to the training plan is also supported by the qualitative feedback from participants during the bi-weekly telephone conversations. For instance, most of the participants frequently engaged in activities other than running or walking-based aerobic endurance exercise, such as cycling, swimming, strength training, or soccer. Furthermore, many participants indicated throughout the unsupervised training period that they did not want to or were unable to engage in interval training or fartlek and, consequently, did not incorporate these into their routines. Overall, both the training documentation via online training diary and feedback from the regularly conducted telephone conversations indicated that the majority of participants did not strictly adhere to the guidelines for training intensity, volume, frequency, or type of physical activity, either because they could not, due to diseases or injuries not related to the intervention or because they did not want to. Finally, the training documentation itself must also be questioned, as many participants did not, as prescribed, record entries in their online training diary immediately after each session. Instead, they did so only (sometimes upon request) several days to weeks after a training session and then documented multiple sessions at once. This undermines the credibility of the quantitative training load data, especially in terms of reported training intensities and thus calculated training load.

### Outcome description: ITT, PP and AT analyses approach

#### Self-rated symptom-related and psychosocial health parameters

In the ITT analysis considering the self-rated symptom-related and psychosocial parameters, 11 out of 14 parameters showed a positive direction of DID (presented with a “+”), indicating a direction of effect consistent to a positive DID ((T1-T0 intervention group) - (T1-T0 control group)) with a stronger positive difference (T1-T0) in the intervention group for parameters where higher values represent better health/wellbeing/fitness or consistent to a negative DID with a stronger negative difference in the intervention group for parameters where lower values represent better health/wellbeing/fitness (Table 3). The strongest SD-standardized DID was observed in headache with an effect of almost one SD. The absolute DID in headache was − 2.54 (− 6.05, 0.98) points as a result of an improvement between T0 and T1 by 2.57 points (range: 0–10 points) on the rating scale in the intervention group and an almost constant average headache symptom rating in the control group. DID of the other parameters with suggested positive direction of DID were even weaker with non-existent to moderate SD-standardized effect sizes (Table 3).

The PP analysis was consistent with the direction of DID estimates compared to the ITT-analysis in most parameters and effect sizes where slightly stronger in almost all parameters with a suggested positive direction of DID compared to the ITT analysis (Table 5). Comparing the AT analysis with the PP analysis results, direction of DID remained unchanged in most parameters and SD-standardized DIDs were slightly lower (Table 6).

**Table 5:** Baseline (T0) and follow-up (T1) means and standard deviation (SD) in intervention and control group, difference between T1 and T0 within groups, difference in differences of intervention and control group with corresponding 95% confidence intervals (95%-CI) of self-rated symptom-related and psychosocial parameters in per-protocol analysis.

Parameter name (range)	Intervention baseline (T0) Mean ± SD	Control baseline (T0) Mean ± SD	Intervention follow-up (T1) Mean ± SD	Control follow-up (T1) Mean ± SD	Difference intervention T1-T0	Difference control T1-T0	Difference in differences (DID) T1-T0 [95%-CI]	Difference in differences (DID) T1-T0 [95%-CI] standardized ^§^	Direction of DID ^$^
N	14	23	14	23					
Current health status (1–5)	2.86 ± 0.77	2.96 ± 0.77	2.21 ± 0.70	2.77 ± 0.92	− 0.64	− 0.18	− 0.46 [− 1.23,0.32]	− 0.60 [− 1.62,0.42]	+
Quality of life (1–4)	3.57 ± 0.51	3.48 ± 0.51	3.64 ± 0.50	3.55 ± 0.60	0.07	0.07	0.00 [− 0.51,0.52]	0.01 [− 1.01,1.02]	−
Satisfaction in general (1–4)	3.29 ± 0.47	3.22 ± 0.60	2.93 ± 0.73	3.00 ± 0.76	− 0.36	− 0.22	− 0.14 [− 0.77,0.49]	− 0.26 [− 1.40,0.89]	−
Satisfaction with health (1–4)	3.21 ± 0.58	3.00 ± 0.52	3.14 ± 0.53	3.19 ± 0.75	− 0.07	0.19	− 0.26 [− 0.85,0.33]	− 0.48 [− 1.56,0.60]	−
Movement competence* (1–4)	3.00 ± 0.56	2.70 ± 0.84	3.16 ± 0.54	2.64 ± 0.74	0.16	− 0.05	0.22 [− 0.48,0.92]	0.29 [− 0.64,1.21]	+
Control competence* (1–4)	2.60 ± 0.62	2.41 ± 0.66	2.91 ± 0.50	2.50 ± 0.62	0.31	0.09	0.22 [− 0.38,0.81]	0.34 [− 0.59,1.26]	+
Self− regulation competence* (1–4)	2.94 ± 0.52	2.75 ± 0.64	3.05 ± 0.46	2.58 ± 0.65	0.11	− 0.17	0.28 [− 0.30,0.85]	0.47 [− 0.50,1.44]	+
Symptoms of depression^†^ (0–45)	8.58 ± 5.62	11.27 ± 8.23	8.94 ± 8.21	12.04 ± 7.12	0.36	0.78	− 0.42 [− 7.57,6.74]	− 0.06 [− 1.03,0.92]	−
Fatigue^#^ (0–10)	4.82 ± 1.89	4.85 ± 2.03	3.10 ± 1.97	4.52 ± 1.94	− 1.72	− 0.33	− 1.39 [− 3.51,0.72]	− 0.71 [− 1.80,0.37]	+
Concentration disorders^#^ (0–10)	4.50 ± 2.27	4.32 ± 2.47	3.20 ± 1.93	4.00 ± 2.42	− 1.30	− 0.32	− 0.98 [− 3.71,1.74]	− 0.41 [− 1.56,0.73]	+
Difficulty breathing^#^ (0–10)	4.17 ± 2.64	4.31 ± 2.29	3.20 ± 1.64	3.64 ± 1.82	− 0.97	− 0.66	− 0.30 [− 3.38,2.78]	− 0.13 [− 1.45,1.19]	+
Headache^#^ (0–10)	4.67 ± 3.06	4.11 ± 2.67	1.75 ± 0.96	4.23 ± 2.83	− 2.92	0.12	− 3.04 [− 7.82,1.74]	− 1.30 [− 2.97,0.66]	+
Current physical performance (0–10)	6.14 ± 1.03	5.30 ± 1.87	6.93 ± 1.64	5.57 ± 1.62	0.79	0.26	0.52 [− 1.02,2.07]	0.32 [− 0.62,1.26]	+
Sleep quality (1–3)	2.00 ± 0.55	2.36 ± 0.58	1.93 ± 0.73	2.29 ± 0.64	− 0.07	− 0.08	0.01 [− 0.60,0.61]	0.01 [− 1.02,1.04]	−

Current health status (1–5 scale with 1 representing “very good” and 5 “bad”), quality of life (1–4 scale with 1 meaning “very bad” and 4 “good”), satisfaction with life in general (1–4 scale with 1 representing “very dissatisfied” and 4 “very satisfied”), satisfaction with health (1–4 scale with 1 meaning “very bad” and 4 “good”), *Sub-competence of physical activity-related health competence (by Pfeifer et al. 2013) presented in mean scale (higher value indicating higher competence), symptoms of depression rated by 15-item Centre for Epidemiologic Studies Depression Scale (CES-D) with a cut-off value of > 17 representing depressive conditions, ^#^current symptoms related to SARS-CoV-2 infection (higher values indicating stronger impairment), current physical performance (0–10 scale with 0 representing “very restricted” and 10 representing “very powerful”), sleep quality ( 1–3 scale with 1 meaning “very good” and 3 “very bad”), ^§^ Difference in difference (DID) per standard deviation (SD) of the analysis population at study baseline (T0), ^$^ “+” means direction of effect consistent to a positive DID ((T1-T0 intervention group) - (T1-T0 control group)) with a stronger positive difference (T1-T0) in the intervention group for parameters where higher values represent better health/wellbeing/fitness or consistent to a negative DID with a stronger negative difference in the intervention group for parameters where lower values represent better health/wellbeing/fitness and “−” direction of effect consistent to a positive DID ((T1-T0 intervention group) - (T1-T0 control group)) with a stronger positive difference (T1-T0) in the intervention group for parameters where higher values represent better health/wellbeing/fitness or consistent to a negative DID with a stronger negative difference in the intervention group for parameters where lower values represent better health/wellbeing/fitness.

**Table 6:** Baseline (T0) and follow-up (T1) means and standard deviation (SD) in intervention and control group, difference between T1 and T0 within groups, difference in differences of intervention and control group with corresponding 95% confidence intervals (95%-CI) of self-rated symptom-related and psychosocial parameters in as-treated analysis.

Parameter name (range)	Intervention baseline (T0) Mean ± SD	Control baseline (T0) Mean ± SD	Intervention follow-up (T1) Mean ± SD	Control follow-up (T1) Mean ± SD	Difference intervention T1-T0	Difference control T1-T0	Difference in differences (DID) T1-T0 [95%-CI]	Difference in differences (DID) T1-T0 [95%-CI] standardized ^§^	Direction of DID^$^
N	17	26	17	26					
Current health status (1–5)	2.76 ± 0.75	2.96 ± 0.77	2.29 ± 0.69	2.80 ± 0.91	− 0.47	− 0.16	− 0.31 [− 1.01,0.39]	− 0.41 [− 1.33,0.52]	+
Quality of life (1–4)	3.59 ± 0.51	3.46 ± 0.51	3.71 ± 0.47	3.48 ± 0.65	0.12	0.02	0.10 [− 0.38,0.58]	0.20 [− 0.76,1.15]	+
Satisfaction in general (1–4)	3.24 ± 0.44	3.15 ± 0.61	2.94 ± 0.66	2.96 ± 0.73	− 0.29	− 0.19	− 0.10 [− 0.66,0.46]	− 0.18 [− 1.20,0.84]	−
Satisfaction with health (1–4)	3.24 ± 0.56	2.96 ± 0.53	3.24 ± 0.56	3.13 ± 0.74	0.00	0.16	− 0.16 [− 0.70,0.38]	− 0.30 [− 1.27,0.68]	−
Movement competence* (1–4)	2.98 ± 0.59	2.69 ± 0.79	3.16 ± 0.58	2.65 ± 0.71	0.18	− 0.04	0.22 [− 0.40,0.85]	0.31 [− 0.55,1.17]	+
Control competence* (1–4)	2.61 ± 0.59	2.44 ± 0.67	2.92 ± 0.47	2.50 ± 0.60	0.31	0.06	0.25 [− 0.29,0.79]	0.39 [− 0.45,1.23]	+
Self-regulation competence* (1–4)	2.94 ± 0.52	2.71 ± 0.62	3.10 ± 0.48	2.55 ± 0.64	0.16	− 0.16	0.32 [− 0.21,0.84]	0.54 [− 0.35,1.42]	+
Symptoms of depression^†^ (0–45)	8.12 ± 5.49	11.59 ± 8.33	8.42 ± 7.89	11.88 ± 7.27	0.30	0.29	0.01 [− 6.53, 6.55]	0.00 [− 0.88,0.88]	−
Fatigue^#^ (0–10)	4.50 ± 2.11	4.59 ± 2.11	3.33 ± 2.39	4.50 ± 1.93	− 1.17	− 0.09	− 1.08 [− 3.19,1.04]	− 0.52 [− 1.53,0.50]	+
Concentration disorders^#^ (0–10)	4.10 ± 2.18	4.19 ± 2.46	3.23 ± 1.92	4.06 ± 2.36	− 0.87	− 0.13	− 0.73 [− 3.16,1.69]	− 0.31 [− 1.35,0.72]	+
Difficulty breathing^#^ (0–10)	4.00 ± 2.62	4.20 ± 2.14	3.86 ± 2.27	3.75 ± 1.95	− 0.14	− 0.45	0.31 [− 2.47,3.08]	0.14 [− 1.09,1.36]	−
Headache^#^ (0–10)	4.67 ± 3.06	4.11 ± 2.67	1.80 ± 0.84	4.21 ± 2.72	− 2.87	0.10	− 2.97 [− 7.39,1.45]	− 1.13 [− 2.81,0.55]	+
Current physical performance (0–10)	6.35 ± 1.06	5.23 ± 1.86	6.94 ± 1.60	5.58 ± 1.77	0.59	0.35	0.24 [− 1.21,1.69]	0.14 [− 0.72,1.01]	+
Sleep quality (1–3)	2.00 ± 0.50	2.32 ± 0.56	1.88 ± 0.70	2.25 ± 0.61	− 0.12	− 0.07	− 0.05 [− 0.57,0.48]	− 0.09 [− 1.04,0.87]	+

Current health status (1–5 scale with 1 representing “very good” and 5 “bad”), quality of life (1–4 scale with 1 meaning “very bad” and 4 “good”), satisfaction with life in general (1–4 scale with 1 representing “very dissatisfied” and 4 “very satisfied”), satisfaction with health (1–4 scale with 1 meaning “very bad” and 4 “good”), *Sub-competence of physical activity-related health competence (by Pfeifer et al. 2013) presented in mean scale (higher value indicating higher competence), symptoms of depression rated by 15-item Centre for Epidemiologic Studies Depression Scale (CES-D) with a cut-off value of > 17 representing depressive conditions, ^#^current symptoms related to SARS-CoV-2 infection (higher values indicating stronger impairment), current physical performance (0–10 scale with 0 representing “very restricted” and 10 representing “very powerful”), sleep quality ( 1–3 scale with 1 meaning “very good” and 3 “very bad”), ^§^ Difference in difference (DID) per standard deviation (SD) of the analysis population at study baseline (T0), ^$^ “+” means direction of effect consistent to a positive DID ((T1-T0 intervention group) - (T1-T0 control group)) with a stronger positive difference (T1-T0) in the intervention group for parameters where higher values represent better health/wellbeing/fitness or consistent to a negative DID with a stronger negative difference in the intervention group for parameters where lower values represent better health/wellbeing/fitness and “−” direction of effect consistent to a positive DID ((T1-T0 intervention group) - (T1-T0 control group)) with a stronger positive difference (T1-T0) in the intervention group for parameters where higher values represent better health/wellbeing/fitness or consistent to a negative DID with a stronger negative difference in the intervention group for parameters where lower values represent better health/wellbeing/fitness.

#### Spiroergometric parameters

In the ITT analysis of spiroergometric parameters 11 out of 15 parameters showed a positive direction of DID (Table 7). Very low to moderate SD-standardized DID were indicated with the strongest SD-standardized DID in heart rate at the thresholds and in heart rate recovery.

**Table 7:** Baseline (T0) and follow-up (T1) means and standard deviation (SD) in intervention and control group, difference between T1 and T0 within groups, difference in differences of intervention and control group with corresponding 95% confidence intervals (95%-CI) of spiroergometric and body composition parameters in the intention-to-treat analysis.

Parameter name (range)	Intervention baseline (T0) Mean ± SD	Control baseline (T0) Mean ± SD	Intervention follow-up (T1) Mean ± SD	Control follow-up (T1) Mean ± SD	Difference intervention T1-T0	Difference control T1-T0	Difference in differences (DID) T1-T0 [95%-CI)	Difference in differences (DID) T1-T0 [95%-CI) standardized^§^	Direction of DID^$^
N	26	28	26	28					
VO2peak relative (ml/min/ kg body weight)	27.69 ± 5.62	26.39 ± 9.11	28.48 ± 6.16	27.43 ± 8.90	0.79	1.04	− 0.25 [− 6.15,5.65]	− 0.03 [− 0.81,0.74]	**−**
VE/VCO2-Slope	29.96 ± 7.18	31.49 ± 6.15	29.75 ± 4.62	30.279 ± 4.62	− 0.21	− 1.21	1.00 [− 3.41,5.41]	0.13 [− 0.45,0.71]	**−**
Peak power (Watt)	187.81 ± 48.18	169.57 ± 56.63	192.68 ± 52.61	172.29 ± 55.97	4.87	2.71	2.16 [− 38.98,43.29]	0.04 [− 0.73,0.82]	+
Peak heart rate (beats/min)	164.15 ± 20.06	157.93 ± 15.96	163.84 ± 18.35	160.96 ± 16.75	− 0.31	3.04	− 3.35 [− 17.01,10.31]	− 0.18 [− 0.94,0.57]	+
Peak breathing rate (breaths/min)	41.54 ± 5.29	40.54 ± 7.07	43.88 ± 12.59	41.96 ± 7.13	2.34	1.43	0.91 [− 5.52,7.34]	0.15 [− 0.89,1.18]	+
Peak respiratory exchange ratio (RER)	1.14 ± 0.05	1.14 ± 0.07	1.15 ± 0.08	1.13 ± 0.07	0.01	− 0.01	0.02 [− 0.04,0.07]	0.27 [− 0.55,1.10]	+
Power ventilatory threshold 1 (Watt)	97.35 ± 22.17	85.30 ± 38.09	99.52 ± 35.21	86.52 ± 34.97	2.17	1.22	0.95 [− 24.79,26.70]	0.03 [− 0.78,0.84]	+
Heart rate ventilatory threshold 1 (beats/min)	117.27 ± 18.97	112.93 ± 15.51	114.96 ± 14.17	115.26 ± 13.57	− 2.31	2.33	− 4.64 [− 16.80,7.51]	− 0.27 [− 0.97,0.44]	+
Power ventilatory threshold 2 (Watt)	152.72 ± 39.34	138.20 ± 48.68	156.08 ± 40.54	139.29 ± 38.95	3.36	1.09	2.27 [− 31.33,35.87]	0.05 [− 0.71,0.81]	+
Heart rate ventilatory threshold 2 (beats/min)	145.60 ± 21.02	140.36 ± 15.70	144.40 ± 17.74	143.00 ± 16.45	− 1.20	2.64	− 3.84 [− 18.09,10.41]	− 0.21 [− 0.98,0.56]	+
Power 2 mmol lactate threshold (Watt)	112.50 ± 42.56	101.89 ± 44.95	111.32 ± 40.54	105.48 ± 44.04	− 1.18	3.60	− 4.78 [− 38.63,29.08]	− 0.11 [− 0.88,0.67]	**−**
Heart rate 2 mmol lactate threshold (beats/min)	124.81 ± 16.75	122.73 ± 15.06	118.68 ± 14.96	122.96 ± 14.64	− 6.13	0.23	− 6.36 [− 18.45,5.74]	− 0.40 [− 1.17,0.36]	+
Power 4 mmol lactate threshold (Watt)	154.96 ± 41.49	141.44 ± 43.77	155.36 ± 38.78	143.54 ± 42.01	0.40	2.09	− 1.69 [− 34.24,30.86]	− 0.04 [− 0.80,0.72]	−
Heart rate 4 mmol lactate threshold (beats/min)	146.76 ± 18.05	142.33 ± 14.02	142.08 ± 16.96	144.58 ± 14.08	− 4.68	2.24	− 6.92 [− 19.31,5.46]	− 0.43 [− 1.20,0.34]	+
Heart Rate Recovery (HRR2) (beats/min)	− 10.92 ± 8.06	− 12.27 ± 5.36	− 13.20 ± 4.36	− 11.866 ± 6.67	− 2.28	0.41	− 2.69 [− 7.59,2.22]	− 0.40 [− 1.12,0.33]	+
Weight (kg)	87.10 ± 16.50	84.02 ± 22.95	84.91 ± 16.50	84.17 ± 23.51	− 2.19	0.15	− 2.34 [− 17.98,13.30]	− 0.12 [− 0.90,0.67]	+
BMI (kg/m^2^)	27.82 ± 3.96	27.80 ± 6.42	27.00 ± 3.55	28.35 ± 6.66	− 0.82	0.54	− 1.36 [− 5.52,2.80]	− 0.26 [− 1.04,0.53]	+
Skeletal muscle mass (kg)	34.64 ± 7.82	31.64 ± 7.66	33.80 ± 7.82	31.39 ± 7.72	− 0.84	− 0.25	− 0.59 [− 6.66,5.47]	− 0.11 [− 0.85,0.70]	−
Body fat mass (kg)	25.39 ± 8.75	26.69 ± 14.75	23.93 ± 8.40	27.23 ± 15.31	− 1.46	0.54	− 1.99 [− 11.65,7.66]	− 0.16 [− 0.96,0.63]	+

$$\dot {V}$$O2peak and RERpeak were defined as the highest 30-second rolling averages during the test, Peak power was calculated as follows: Wf + [(t/D x P)], where Wf represents the value of the last completed workload (W), t is the time (s) the last uncompleted workload was sustained, D is the duration (s) of each stage, and P is the power output difference between workloads, Watt and heart rate at ventilator threshold 1 and ventilator threshold 2 were determined visually by combining four methods: the ventilatory equivalent method, the excess carbon dioxide method, the V-slope method, and the end-tidal method. ^§^ Difference in difference (DID) per standard deviation (SD) of the analysis population at study baseline (T0),^$^“+” means direction of effect consistent to a positive DID ((T1-T0 intervention group) - (T1-T0 control group)) with a stronger positive difference (T1-T0) in the intervention group for parameters where higher values represent better fitness or consistent to a negative DID with a stronger negative difference in the intervention group for parameters where lower values represent better fitness and “−” direction of effect consistent to a positive DID ((T1-T0 intervention group) - (T1-T0 control group)) with a stronger positive difference (T1-T0) in the intervention group for parameters where higher values represent better fitness or consistent to a negative DID with a stronger negative difference in the intervention group for parameters where lower values represent better fitness.

In PP analysis, most parameters showed a direction of DID consistent to the ITT analysis. No clear changes in SD-standardised DID compared to the ITT analysis could be observed (Table 8). The direction of DID in the AT analysis was consistent to the PP analysis and DID only changed slightly with the strongest changes in peak power (DID: 9.56 [-35.16, 54.28] and power at 2 mmol lactate threshold (DID: 10.02 [-28.90, 48.94]) (Table 9).

**Table 8:** Baseline (T0) and follow-up (T1) means and standard deviation (SD) in intervention and control group, difference between T1 and T0 within groups, difference in differences of intervention and control group with corresponding 95% confidence intervals (95%-CI) of spiroergometric and body composition parameters in per-protocol analysis.

Parameter name (unit)	Intervention baseline (T0) Mean ± SD	Control baseline (T0) Mean ± SD	Intervention follow-up (T1) Mean ± SD	Control follow-up (T1) Mean ± SD	Difference intervention T1-T0	Difference control T1-T0	Difference in differences (DID) T1-T0 [95%-CI]	Difference in differences (DID) T1-T0 [95%-CI] standardized ^§^	Direction of DID^$^
N	14	23	14	23					
VO2peak relative (ml/min/ kg body weight)	28.86 ± 6.32	26.09 ± 8.64	30.29 ± 6.92	26.44 ± 8.26	1.43	0.35	1.08 [− 6.40,8.56]	0.14 [− 0.81,1.09]	+
VE/VCO2-Slope	28.16 ± 8.33	31.64 ± 6.38	29.01 ± 3.47	29.95 ± 4.53	0.85	− 1.69	2.54 [− 3.066,8.15]	0.35 [− 0.42,1.12]	−
Peak power (Watt)	187.00 ± 47.37	168.44 ± 56.32	194.00 ± 47.12	168.70 ± 53.32	7.00	0.26	6.74 [− 43.12,56.60]	0.13 [− 0.81,1.06]	+
Peak heart rate (beats/min)	167.79 ± 21.33	160.09 ± 14.66	166.71 ± 16.50	162.04 ± 15.44	− 1.07	1.96	− 3.03 [− 18.96,12.90]	− 0.17 [− 1.08,0.73]	+
Peak breathing rate (breaths/min)	41.21 ± 5.48	40.22 ± 7.24	41.29 ± 5.77	41.35 ± 7.23	0.07	1.13	− 1.06 [− 7.45,5.33]	− 0.16 [− 1.13,0.81]	−
Peak respiratory exchange ratio (RER)	1.16 ± 0.05	1.15 ± 0.07	1.14 ± 0.08	1.13 ± 0.07	− 0.02	− 0.01	− 0.00 [− 0.07,0.06]	− 0.06 [− 1.08,0.96]	−
Power ventilatory threshold 1 (Watt)	96.36 ± 22.85	82.32 ± 37.63	97.36 ± 31.89	86.77 ± 35.22	1.00	4.46	− 3.45 [− 35.67,28.76]	− 0.10 [− 1.08,0.87]	−
Heart rate ventilatory threshold 1 (beats/min)	121.00 ± 21.04	113.00 ± 14.68	117.00 ± 16.33	115.73 ± 13.51	− 4.00	2.73	− 6.73 [− 22.24,8.78]	− 0.38 [− 1.26,0.50]	+
Power ventilatory threshold 2 (Watt)	148.79 ± 33.79	134.67 ± 46.71	157.71 ± 36.99	139.85 ± 38.98	8.93	5.18	3.75 [− 35.64,43.14]	0.09 [− 0.85,1.03]	+
Heart rate ventilatory threshold 2 (beats/min)	147.36 ± 22.53	140.95 ± 14.27	150.00 ± 17.44	142.45 ± 16.39	2.64	1.50	1.15 [− 15.92,18.21]	0.06 [− 0.88,1.01]	−
Power 2 mmol lactate threshold (Watt)	111.43 ± 44.20	101.67 ± 49.10	117.71 ± 39.68	104.75 ± 45.64	6.29	3.08	3.20 [− 41.21,47.61]	0.07 [− 0.88,1.02]	+
Heart rate 2 mmol lactate threshold (beats/min)	130.43 ± 14.97	123.48 ± 15.83	125.07 ± 13.02	123.80 ± 15.33	− 5.36	0.32	− 5.68 [− 20.35,9.00]	− 0.36 [− 1.30,0.57]	+
Power 4 mmol lactate threshold (Watt)	153.43 ± 38.95	139.39 ± 44.79	157.86 ± 36.29	142.71 ± 43.13	4.43	3.32	1.11 [− 39.13,41.34]	0.03 [− 0.92,0.97]	+
Heart rate 4 mmol lactate threshold (beats/min)	150.50 ± 16.72	143.65 ± 13.67	147.21 ± 14.83	145.62 ± 13.39	− 3.29	1.97	− 5.25 [− 19.20,8.69]	− 0.35 [− 1.28,0.58]	+
Heart rate recovery (HRR2) (beats/min)	− 12.00 ± 7.97	− 12.32 ± 5.09	− 14.40 ± 3.78	− 12.14 ± 7.15	− 2.40	0.19	− 2.59 [− 8.54,3.36]	− 0.42 [− 1.37,0.54]	+
Weight (kg)	83.99 ± 17.07	85.93 ± 23.82	82.54 ± 17.09	86.03 ± 24.76	− 1.44	0.10	− 1.54 [− 22.52,19.45]	− 0.07 [− 1.06,0.91]	+
BMI (kg/m^2^)	27.50 ± 3.98	28.34 ± 6.74	27.00 ± 3.86	28.93 ± 7.03	− 0.50	0.59	− 1.09 [− 6.82,4.63]	− 0.19 [− 1.18,0.80]	+
Skeletal muscle mass (kg)	33.34 ± 7.58	32.06 ± 7.86	32.32 ± 7.37	31.62 ± 8.02	− 1.01	− 0.44	− 0.58 [− 8.20,7.04]	− 0.08 [− 1.07,0.92]	−
Body fat mass (kg)	24.45 ± 9.95	27.93 ± 15.76	23.32 ± 9.75	28.66 ± 16.41	− 1.13	0.73	− 1.85 [− 15.64,11.93]	− 0.13 [− 1.14,0.87]	+

$$\dot {V}$$O2peak and RERpeak were defined as the highest 30-second rolling averages during the test, Peak power was calculated as follows: Wf + [(t/D × P)], where Wf represents the value of the last completed workload (W), t is the time (s) the last uncompleted workload was sustained, D is the duration (s) of each stage, and P is the power output difference between workloads, Watt and heart rate at ventilator threshold 1 and ventilator threshold 2 were determined visually by combining four methods: the ventilatory equivalent method, the excess carbon dioxide method, the V-slope method, and the end-tidal method. ^§^ Difference in difference (DID) per standard deviation (SD) of the analysis population at study baseline (T0),^$^ “+” means direction of effect consistent to a positive DID ((T1-T0 intervention group) - (T1-T0 control group)) with a stronger positive difference (T1-T0) in the intervention group for parameters where higher values represent better fitness or consistent to a negative DID with a stronger negative difference in the intervention group for parameters where lower values represent better fitness and “−” direction of effect consistent to a positive DID ((T1-T0 intervention group) - (T1-T0 control group)) with a stronger positive difference (T1-T0) in the intervention group for parameters where higher values represent better fitness or consistent to a negative DID with a stronger negative difference in the intervention group for parameters where lower values represent better fitness.

**Table 9:** Baseline (T0) and follow-up (T1) means and standard deviation (SD) in intervention and control group, difference between T1 and T0 within groups, difference in differences of intervention and control group with corresponding 95% confidence intervals (95%-CI) of spiroergometric and body composition parameters in as-treated analysis.

Parameter name (unit)	Intervention baseline (T0) Mean ± SD	Control baseline (T0) Mean ± SD	Intervention follow-up (T1) Mean ± SD	Control follow-up (T1) Mean ± SD	Difference intervention T1-T0	Difference control T1-T0	Difference in differences (DID) T1-T0 [95%-CI]	Difference in differences (DID) T1-T0 [95%-CI] standardized ^§^	Direction of DID ^$^
N	17	26	17	26					
VO2peak relative (ml/min/ kg body weight)	29.53 ± 7.75	25.73 ± 8.23	31.29 ± 8.25	26.08 ± 7.92	1.77	0.35	1.42 [− 5.64,8.48]	0.17 [− 0.69,1.04]	+
VE/VCO2-Slope	28.19 ± 7.51	32.01 ± 6.41	28.98 ± 3.54	29.97 ± 4.68	0.79	− 2.04	2.83 [− 2.18,7.85]	0.40 [− 0.31,1.12]	−
Peak power (Watt)	192.18 ± 46.65	166.35 ± 53.54	200.47 ± 49.62	165.08 ± 51.81	8.29	− 1.27	9.56 [− 35.16,54.28]	0.18 [− 0.68,1.04]	+
Peak heart rate (beats/min)	164.53 ± 22.73	159.12 ± 15.36	164.18 ± 19.39	161.39 ± 15.52	− 0.35	2.27	− 2.62 [− 18.31,13.06]	− 0.14 [− 0.99,0.70]	+
Peak breathing rate (breaths/min)	41.12 ± 5.00	40.23 ± 6.92	41.65 ± 5.61	40.89 ± 6.99	0.53	0.65	− 0.12 [− 5.71,5.46]	− 0.02 [− 0.92,0.88]	−
Peak respiratory exchange ratio (RER)	1.15 ± 0.06	1.15 ± 0.07	1.13 ± 0.08	1.14 ± 0.07	− 0.02	− 0.01	− 0.01 [− 0.07,0.05]	− 0.11 [− 1.06,0.84]	−
Power ventilatory threshold 1 (Watt)	101.29 ± 25.73	83.64 ± 35.68	99.82 ± 29.55	84.08 ± 34.39	− 1.47	0.44	− 1.91 [− 30.49,26.67]	− 0.06 [− 0.93,0.81]	−
Heart rate ventilatory threshold 1 (beats/min)	120.29 ± 20.51	112.52 ± 16.13	115.71 ± 16.62	114.48 ± 13.80	− 4.59	1.96	− 6.55 [− 21.21,8.11]	− 0.36 [− 1.17,0.45]	+
Power ventilatory threshold 2 (Watt)	154.53 ± 36.92	132.74 ± 45.15	159.63 ± 35.45	136.61 ± 39.03	5.10	3.87	1.22 [− 34.99,37.44]	0.03 [− 0.82,0.88]	+
Heart rate ventilatory threshold 2 (beats/min)	145.35 ± 23.31	140.35 ± 15.27	149.00 ± 18.17	141.52 ± 16.30	3.65	1.17	2.47 [− 14.00,18.94]	0.13 [− 0.74,1.00]	−
Power 2 mmol lactate threshold (Watt)	112.41 ± 40.02	99.75 ± 47.65	121.29 ± 36.66	98.61 ± 45.59	8.88	− 1.14	10.02 [− 28.90,48.94]	0.22 [− 0.65,1.10]	+
Heart rate 2 mmol lactate threshold (beats/min)	127.47 ± 15.57	121.29 ± 16.51	123.82 ± 13.48	120.96 ± 16.73	− 3.65	− 0.34	− 3.31 [− 17.48,10.85]	− 0.20 [− 1.08,0.67]	+
Power 4 mmol lactate threshold (Watt)	156.94 ± 36.11	136.44 ± 44.19	161.24 ± 33.62	138.67 ± 42.21	4.29	2.23	2.07 [− 33.53,37.67]	0.05 [− 0.80,0.90]	+
Heart rate 4 mmol lactate threshold (beats/min)	147.18 ± 17.90	142.28 ± 15.13	145.29 ± 15.91	144.08 ± 15.23	− 1.88	1.80	− 3.69 [− 17.83,10.46]	− 0.23 [− 1.10,0.64]	+
Heart Rate Recovery (HRR2) (beats/min)	− 12.41 ± 7.70	− 11.63 ± 6.00	− 14.15 ± 3.58	− 11.94 ± 6.90	− 1.74	− 0.30	− 1.43 [− 7.03,4.16]	− 0.22 [− 1.06,0.63]	+
Weight (kg)	84.14 ± 16.50	85.33 ± 22.53	82.99 ± 16.35	85.19 ± 23.47	− 1.15	− 0.14	− 1.01 [− 19.21,17.19]	− 0.05 [− 0.95,0.85]	+
BMI (kg/m^2^)	27.42 ± 4.11	28.09 ± 6.36	27.02 ± 3.98	28.55 ± 6.70	− 0.40	0.45	− 0.85 [− 5.87,4.17]	− 0.15 [− 1.06,0.76]	+
Skeletal muscle mass (kg)	33.59 ± 6.98	31.90 ± 7.49	32.90 ± 6.78	31.50 ± 7.62	− 0.69	− 0.40	− 0.29 [− 6.83,6.25]	− 0.04 [− 0.94,0.86]	−
Body fat mass (kg)	24.14 ± 9.81	27.58 ± 14.78	23.06 ± 9.63	28.05 ± 15.41	− 1.08	0.46	− 1.54 [− 13.40,10.32]	− 0.12 [− 1.03,0.80]	+

$$\dot {V}$$O2peak and RERpeak were defined as the highest 30-second rolling averages during the test, Peak power was calculated as follows: Wf + [(t/D × P)], where Wf represents the value of the last completed workload (W), t is the time (s) the last uncompleted workload was sustained, D is the duration (s) of each stage, and P is the power output difference between workloads, Watt and heart rate at ventilator threshold 1 and ventilator threshold 2 were determined visually by combining four methods: the ventilatory equivalent method, the excess carbon dioxide method, the V-slope method, and the end-tidal method. ^§^Difference in difference (DID) per standard deviation (SD) of the analysis population at study baseline (T0),^$^ “+” means direction of effect consistent to a positive DID ((T1-T0 intervention group) - (T1-T0 control group)) with a stronger positive difference (T1-T0) in the intervention group for parameters where higher values represent better fitness or consistent to a negative DID with a stronger negative difference in the intervention group for parameters where lower values represent better fitness and “−” direction of effect consistent to a positive DID ((T1-T0 intervention group) - (T1-T0 control group)) with a stronger positive difference (T1-T0) in the intervention group for parameters where higher values represent better fitness or consistent to a negative DID with a stronger negative difference in the intervention group for parameters where lower values represent better fitness.

#### Body composition parameters

In the ITT analysis of the body composition parameters, weight, BMI and body fat mass indicated positive direction of DID, with BMI indicating the strongest SD-standardized DID (− 0.26 [− 1.04, 0.53]) (Table 7). In the PP and AT analysis of the bioelectrical impedance analysis, direction of DID were consistent to ITT analysis with slightly weaker effect sizes (Table 8  and  9).

The proportion (p̂) of parameters with a positive direction of DID was 0.76 (25 out of 33 parameters) in the ITT analysis with a corresponding Clopper-Pearson 95% CI of 0.58 to 0.89. This indicated that the proportion of positive DID may not have been expected by chance. The proportion of positive DID in the PP (p̂ = 0.67 [0.48, 9.82]) and AT (p̂ = 0.70 [0.51, 0.84]) analysis were slightly lower.

## Discussion

Here we report on the limited feasibility of a 12-week unsupervised aerobic exercise training intervention among participants experiencing persistent, moderately severe symptoms > 12 months after SARS-CoV-2 infection. The intervention of the present study proves feasibility in terms of safety and tolerability, as participants did not report any adverse events or safety concerns related to the intervention. However, feasibility is constrained by low adherence to the training plan and incomplete maintenance of the training diary, which limits the ability to draw firm conclusions regarding efficacy. This explorative pilot study used descriptive analyses to inform future research testing the efficacy of an unsupervised aerobic exercise training program.

The present study shows weak indications of a potential reduction in self-rated symptom severity and improvement in overall well-being. Across most investigated parameters including symptom-related, psychosocial, spiroergometric, and body composition outcomes, effect sizes show a positive direction even in the ITT analysis, which represents the most conservative analysis approach. The strongest standardized DID are observed for symptom-related and psychosocial parameters. Nonetheless, all explored group differences remain small to moderate, with low precision of effect size estimates (i.e., wide 95% confidence intervals) due to the small sample size. Several potential factors may contribute to the modest effect sizes in the present study. Due to the interventions aim to prevent initial overstress and exhaustion, the initial training load was kept low. Consequently, participants only reached the recommendations of the World Health Organization (WHO) for physical activity (> 150 min of light to moderate physical activity per week) at the end of the training plan. This initial low training load, defined by intensity by duration, may contribute to the very low effect sizes, particularly in the spiroergometric parameters. Higher training loads, in terms of intensity and/or duration, appear to be necessary to achieve efficacy at the cardiovascular level.

A randomized controlled trial (RCT) by Jimeno-Almazán et al., investigating higher training loads in terms of increased intensities, has indicated more substantial improvements in both psychosocial and cardiovascular fitness-related parameters in the intervention group (*n* = 19) compared to the control group (*n* = 20) in patients with post-COVID-19 condition. The 8-week supervised resistance and endurance training has demonstrated that an interval training twice a week (4–6 × 3–5 min) at a rating of perceived exertion (RPE, according to the 6–20 Borg scale) of 16, coupled with steady-state training (30–60 min) once a week at an RPE of 12, in addition to resistance training, has been safe, well-tolerated, and more effective compared to the control group, which followed the self-management WHO rehabilitation leaflet^38^. Additionally, other supervised intervention studies have indicated that higher training intensities in the form of high-intensity interval training (HIIT) have been safely tolerated and may improve functional capacity in individuals recently hospitalized for severe COVID-19^39,40^. Achieving a higher training load in aerobic exercise training programs could also be possible with longer training durations.

In the present, some participants exceeded the planned training duration, resulting in higher mean training loads compared to the planned training loads in the as-treated (AT) analysis population. The mean difference in relative VO2peak (1,8 ml/kg/min) from baseline to follow-up in the present AT population is comparable to the results of the 8-week supervised intervention by Jimeno-Almazán et al. (2,1 ml/kg/min), despite the unsupervised nature and lower intensity of the present intervention.

The present unsupervised intervention reveals limited compliance, as many participants did not strictly adhere to the training plan and failed to consistently maintain the training diary. In general, supervised interventions facilitate higher compliance, likely due to structured oversight and real-time feedback. Other unsupervised intervention studies involving previously hospitalized COVID-19 patients addressed this by monitoring compliance using intensity measurement tools. For example, Li et al. utilized heart rate telemetry devices, while Kortianou et al. measured heart rate and oxygen saturation^41,42^. Despite the determination of objective load parameters to monitor the compliance, these unsupervised interventions also showed limited efficacy across most of the investigated parameters, comparable to the findings of the present study.

However, in the RCT by Li et al., an unsupervised, multidisciplinary 6-week exercise program has demonstrated superior effects on functional exercise capacity (6-minute walking test), lower limb muscle strength, and physical health-related quality of life in the intervention group (*n* = 59) compared to the control group (*n* = 61). No effects have been observed on pulmonary function parameters and mental health-related quality of life^41^. Similarly, the observational study by Kortianou et al. (*n* = 22), investigated a combination of an unsupervised exercise program (including strength and endurance training) with one-hour supervised tele-rehabilitation sessions every 10 days. This approach resulted in improvements in analyzed psychological parameters, specifically anxiety, depression, and quality of life, but showed limited effects in most physical performance test parameters^42^which is consistent with findings from the present study.

In the here described intervention, no objective intensity measures during the training were employed. Participants were instructed to self-monitor their training intensity exclusively using Borg’s CR10 scale. Although rating of perceived exertion (RPE) is a widely used tool to gauge training intensity, its accurate application and interpretation require practice and experience. Therefore, the utilization of a RPE scale among inexperienced individuals may have therefore compromised the quality and consistency of training, particularly during interval training sessions, and could have led to inefficient training outcomes^33^.

In summary, unsupervised aerobic exercise interventions for individuals with post-COVID-19 condition must ensure a sufficient training load without provoking overstress and exhaustion. Ensuring high compliance in unsupervised interventions is essential, yet appears to be more achievable in supervised interventions. Further high-quality evidence is required to evaluate the efficacy of unsupervised aerobic exercise training programs in individuals with persistent symptoms after SARS-CoV-2 infection. Further studies should place particular emphasis on monitoring both training load and participant compliance.

This present study, designed as a pilot study to explore the adherence to an unsupervised aerobic exercise training program for participants with persistent symptoms > 12 months after SARS-CoV-2 infection, includes a limited sample size. Given the broad range of health-related parameters assessed to generate hypotheses for future studies, the statistical power is insufficient to precisely estimate small to moderate group differences. Additionally, a higher dropout rate among female participants in the intervention group leads to a slight difference in sex distribution after randomization, which may affect the comparability of groups.

Another limitation is the absence of an objective measurement tool for the adherence to the predefined exercise intensity in the unsupervised training setting. Other feasibility domains (e.g., recruitment rate, retention, outcome completion, tolerability) were not comprehensively evaluated. There may have been potential isomorphism due to the use of single questions to measure aspects of quality of life in the present analysis. For some participants, the interval between baseline and follow-up examinations exceeds 12 weeks, potentially influencing the consistency and comparability of results. This extended interval is primarily due to acute illness, injuries, or participants’ difficulties in scheduling the follow-up (T1) assessment in time.

This study indicates limited feasibility of a 12-week unsupervised aerobic exercise training intervention for participants with persistent symptoms of moderate severity > 12 months after infection with SARS-CoV-2, as a large proportion of participants in the intervention group did not strictly adhere to the training plan. As a result, statements about the efficacy are restricted. The recommendation for an unsupervised aerobic exercise training intervention in this population is therefore limited. Nonetheless, the present findings offer important insights to inform the design of future studies investigating the efficacy of unsupervised aerobic exercise training programs in individuals with persistent symptoms after SARS-CoV-2 infection.

## Supplementary Information

Below is the link to the electronic supplementary material.

Supplementary Material 1

## Data Availability

Due to data security reasons (i.e., data contain potentially participant identifying information), the SPOVID Study does not allow sharing data as a public use file. However, others can access the data used upon request, which is the same way authors of the present paper obtained the data. Data requests can be addressed to: recall@uk-essen.de.
