# Supplementary material for: Feasibility of an unsupervised aerobic exercise training program for participants with persistent symptoms after SARS-CoV-2 infection
Source: Sci Rep. 2025 Aug 6;15:28827. doi: 10.1038/s41598-025-13905-4 (PMC12328756; doi:10.1038/s41598-025-13905-4)
Supplement: Supplementary file 1 — Supplementary Material 1 [file 41598_2025_13905_MOESM1_ESM.pdf]

Supplementary Material

Supplementary Table S1: 12-week-long aerobic endurance training plan.

Week	Training Session	Training Type	Intensity	Volume
1	1	Steady-state	Moderate (3)	20 min
	2	Interval	Strong (5)	3 x 4 min (4 min active recovery between intervals)
	3	Steady-state	Moderate (3)	15 min
2	4	Steady-state	Moderate (3)	25 min
	5	Interval	Strong (5)	3 x 5 min (5 min active recovery between intervals)
	6	Steady-state	Moderate (3)	20 min
3	7	Steady-state	Moderate (3)	30 min
	8	Interval	Strong (5)	3 x 6 min (6 min active recovery between intervals)
	9	Steady-state	Moderate (3)	30 min
4	10	Steady-state	Moderate (3)	20 min
	11	Fartlek	Strong (5)	25 min
	12	Steady-state	Moderate (3)	20 min
5	13	Steady-state	Moderate (3)	35 min
	14	Interval	Strong (5)	4 x 4 min (4 min active recovery between intervals)
	15	Steady-state	Moderate (3)	30 min
6	16	Steady-state	Moderate (3)	40 min
	17	Interval	Strong (5)	4 x 5 min (5 min active recovery between intervals)
	18	Steady-state	Moderate (3)	35 min
7	19	Steady-state	Moderate (3)	45 min
	20	Interval	Strong (5)	4 x 6 min (6 min active recovery between intervals)
	21	Steady-state	Moderate (3)	40 min
8	22	Steady-state	Moderate (3)	25 min
	23	Fartlek	Strong (5)	30 min
	24	Steady-state	Moderate (3)	25 min
9	25	Steady-state	Moderate (3)	55 min
	26	Interval	Strong (5)	5 x 4 min (4 min active recovery between intervals)
	27	Steady-state	Moderate (3)	50 min
10	28	Steady-state	Moderate (3)	60 min
	29	Interval	Strong (5)	5 x 5 min (5 min active recovery between intervals)
	30	Steady-state	Moderate (3)	55 min
11	31	Steady-state	Moderate (3)	65 min
	32	Interval	Strong (5)	5 x 6 (6 min active recovery between intervals)
	33	Steady-state	Moderate (3)	60 min
12	34	Steady-state	Moderate (3)	25 min
	35	Fartlek	Strong (5)	30 min
	36	Steady-state	Moderate (3)	25 min

The intensity is given as the intended number on Borg`s CR10 scale [1] , which corresponds to a range from no exertion whatsoever (0) to most strenuous exertion (10). “Moderate” is somewhat but not especially hard and “strong” is hard and tiring, but continuing is not terribly difficult. The intended intensity for active recovery during interval training was set to be light (2).

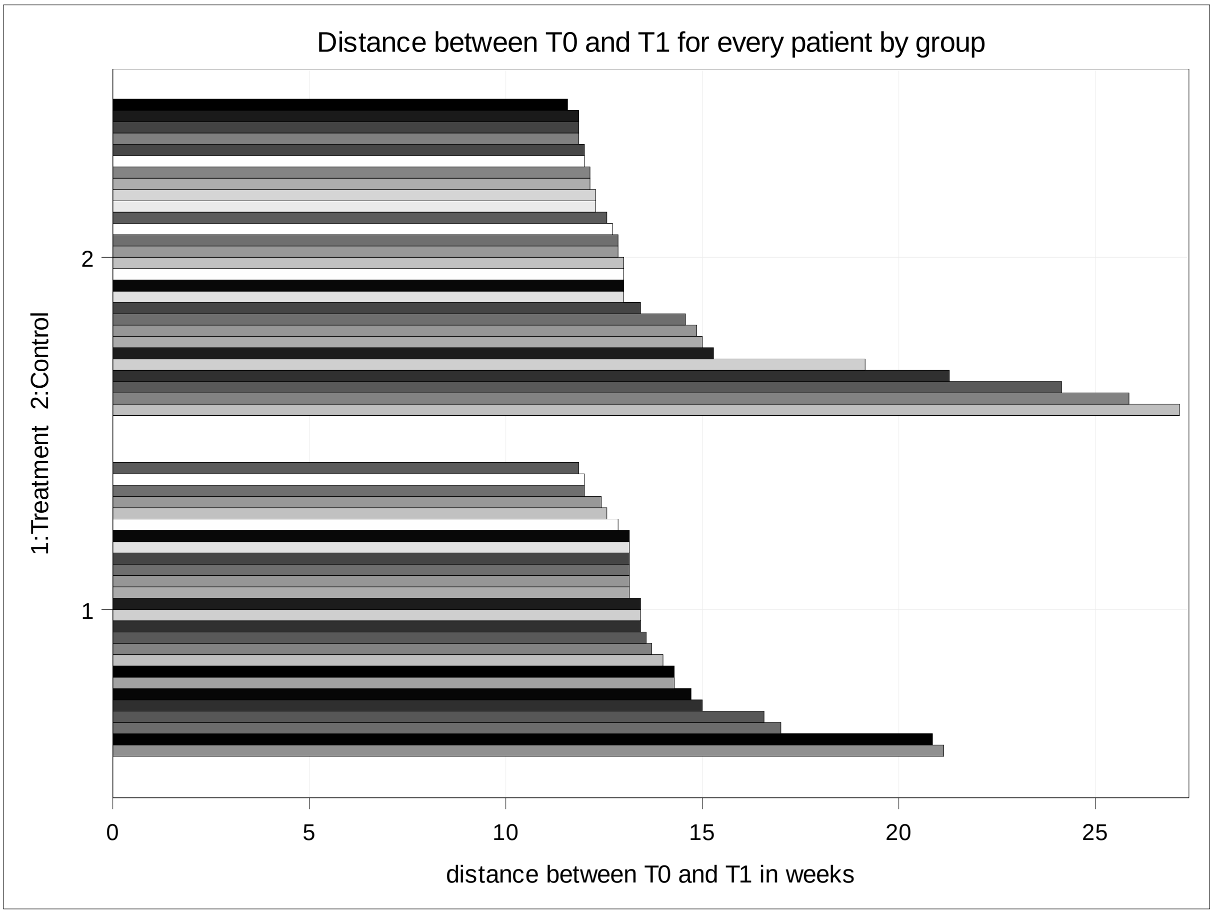

Supplementary Figure S1: Time distance between T0 and T1 for every patient stratified by group

Supplementary Table S2: Intention-to-treat analysis of group-based training load data.

Variable	Planned	Intervention Group	Control Group
		Mean ± SD	
Training Weeks (n)	12	12.8 ± 1.9	12.5 ± 1.2
Total Training Sessions (n)	36	30.4 ± 20.7	25.0 ± 13.0
Weekly Training Sessions (n)	3	2.4 ± 1.7	2.0 ± 1.1
Total Training Duration (h)	20.8	23.7 ± 19.8	22.2 ± 19.1
Weekly Training Duration (h)	1.7	1.9 ± 1.6	1.8 ± 1.5
Average Training Intensity (0-10)*	3.7	3.8 ± 1.0	4.0 ± 1.0
Total Training Load (au)§	4550	5482 ± 4496	5602 ± 5319
Weekly Training Load (au) §	379	428 ± 366	448 ± 440

n=number, h=hours, *intensity as rating of perceived exertion (RPE) using Borg`s CR10 scale [1,2], ranging from no exertion whatsoever (0) to most strenuous exertion (10), §au=arbitrary units, determined multiplying the absolute training duration in minutes by the training intensity [3], SD=standard deviation

Supplementary Table S3: Per-protocol analysis of group-based training load data.

Variable	Planned	Intervention Group	Control Group
		Mean ± SD	
Training Weeks (n)	12	13.2 ± 2.1	12.3 ± 1.3
Total Training Sessions (n)	36	39.9 ± 21.4	6.7 ± 6.9
Weekly Training Sessions (n)	3	3.1 ± 1.8	0.6 ± 0.6
Total Training Duration (h)	20.8	32.7 ± 20.6	8.0 ± 9.5
Weekly Training Duration (h)	1.7	2.6 ± 1.8	0.7 ± 0.8
Average Training Intensity (0-10)*	3.7	4.0 ± 1.0	3.7 ± 0.8
Total Training Load (au)	4550	7835 ± 4466	1991 ± 2535
Weekly Training Load (au)	379	610 ± 378	166 ± 213

n=number, h=hours, *intensity as rating of perceived exertion (RPE) using Borg`s CR10 scale [1,2], ranging from no exertion whatsoever (0) to most strenuous exertion (10), §au=arbitrary units, determined multiplying the absolute training duration in minutes by the training intensity [3], SD=standard deviation

Supplementary Table S4: As-treated analysis of group-based training load data.

Variable	Planned	Intervention Group	Control Group
		Mean ± SD	
Training Weeks (n)	12	13.0 ± 2.0	12.2 ± 1.2
Total Training Sessions (n)	36	40.0 ± 19.5	7.1 ± 6.6
Weekly Training Sessions (n)	3	3.2 ± 1.7	0.6 ± 0.6
Total Training Duration (h)	20.8	36.8 ± 21.9	7.5 ± 9.0
Weekly Training Duration (h)	1.7	2.9 ± 1.8	0.6 ± 0.8
Average Training Intensity (0-10)*	3.7	4.1 ± 1.1	3.8 ± 0.9
Total Training Load (au)	4550	9173 ± 5442	1864 ± 2384
Weekly Training Load (au)	379	728 ± 461	155 ± 200

n=number, h=hours, *intensity as rating of perceived exertion (RPE) using Borg`s CR10 scale [1,2], ranging from no exertion whatsoever (0) to most strenuous exertion (10), §au=arbitrary units, determined multiplying the absolute training duration in minutes by the training intensity [3], SD=standard deviation
